# City size based scaling of the urban internal nodes layout

**DOI:** 10.1371/journal.pone.0250348

**Published:** 2021-04-23

**Authors:** Wenhan Feng, Bayi Li, Zebin Chen, Peng Liu

**Affiliations:** 1 Faculty of Architecture, RWTH Aachen University, Aachen, Germany; 2 School of GeoSciences, University of Edinburgh, Edinburgh, United Kingdom; 3 Department of Architecture, National Univeristy of Singapore, Singapore, Singapore; 4 School of Architecture and Urban Planning, Huazhong University of Science and Technology, Wuhan, China; Institute for Advanced Sustainability Studies, GERMANY

## Abstract

The size of a city is not only essential for depicting the scale of the urban system, but also crucial to support the prosperity, order, and high-speed developments. However, its relation to the underlying urban structure has not been empirically investigated in detail. To examine the impact of city size on the city structure and quantify structural features, in this study, a statistical analysis was performed based on network science and an interdisciplinary theoretical system. To obtain the statistical law of internal node layout, the urban system was regarded as a complete graph weighted by the Euclidean distance. The relationship between the urban internal nodes layout (points of interest data, Weibo check-in data, and central point of road intersection data) and the city size was established. The results confirmed the existence of statistical laws in the layout of urban spatial elements, and explored the relationship between the changes in urban node network structure and inequality. This study provided a new perspective of urban structure to understand the complexity of the city, and suggested an approach to adjust this structure to narrow down the gap between the urban and rural areas.

## Introduction

A city is a complex system consisting of urban road networks and comprehensive functions. The features of a city include multi-dimensionality, randomness, and dynamics. The size of cities is known to play a fundamental role. A power law relation (1) exists between multiple variables and city size.

$$\begin{array}{c} ϒ\propto {\text{N}}^{\beta } \end{array}$$ (1)

where ϒ is a variable, N is a constant, and *β* is the scaling exponent.

The relationship between urban scaling and economies, as well as the optimal size for a city, has been studied for years [1]. Network science links city size to an urban network [2]. Recently, network analyses of city systems have been conducted in the fields of sociology, economics, computer science, and urban planning [3, 4], and their outcomes have been applied to a wide variety of socioeconomic quantities, including wealth, innovation, crime, and investment [5–8].

The complexity and uniqueness of urban structures introduce challenges in elucidating the urban characteristics abstractly. Several studies have observed the features of urban structures, such as the traditional model of urban fracture patterns [9]. The recent availability of new large-scale datasets, such as those from social media check-in data and open street maps, presents unprecedented possibilities to systematically study the urban socioeconomic dynamics. Many convenient datasets have led to a concentration of social networks in the field of urban structure research. Tu et al. proposed a methodology for urban function quantization using the data obtained from mobile phones and social media [10]. Ji et al. applied graph modeling to explore the landmark comments from social media datasets to quantify the popularity of spaces and traffic networks [11]. From another perspective, the automatic smart card data was applied in weighted directed graphs to identify the spatial structure of city hubs, centers, and borders [12, 13]. Boeing selected street networks from OpenStreetMap (OSM), and analyzed the street network characteristics at the metropolitan, municipal, and neighborhood scales [14]. These studies proved the impact of urban structures on development and economic activities, and verified the efficiency of multi-source data in an urban quantitative study. It can be further determined that, similar to the city size, the urban structure also exhibits statistical regularity with many phenomena in the city, such as inequality and pollution [15, 16]. Thus, there exists a logic that enables the structure of the city to be scaled to the corresponding city size, similar to most other cases. Although these studies considered the cases of different countries and years [17], the statistical relationship between the city size and function layout has not been tested empirically in detail yet.

Empirically, as the population increases, the city area expands. Therefore, it can be stated that the urban structures of the cities of similar size have some commonality in their geographical space. Based on a literature review and previous techniques, this study attempts to verify this statement, i.e., the existence of a relationship between the characteristics of the urban structure and city size. In this study, a city was regarded as a complete graph composed of nodes, which was weighted with the relative distance between its nodes. The extraction of the statistical characteristic values of the weighted network enabled the establishment of the relationship between the structure and size of a city. We observed that starting from a certain node in the city, with increasing distance, the probability of nodes existing in the geographic space tended to exhibit a log-normal distribution, while the distance corresponding to the highest probability value was positively correlated with the city size. On this basis, we investigated the correlation of the node layout with urban and rural development inequality.

Through network analysis and node viewpoints, this study analyzed the points of interest (POI) and Weibo check-in data sets(Location Based Services, LBS), as well as the central point of road intersection(CPRI) of several cities. Due to the characteristics of these data, this study focused on the complexity of urban nodes. Further explorations demonstrated the relationship between city scale and network structure of human interaction. The main contributions of this study are described below:

1. Summarized the statistical characteristics of the urban internal node layout, and explored its causes.
2. Verified the relationship between relative distance and population, and defined a quantitative method.
3. Taking the urban-rural gap as an example, analyzed the influence of urban structure on the urban power law, and suggested feasible strategy to narrow urban-rural gap by adjusting the urban structure. And deduced the influence of urban structure on urban development.

## Experimental data and methods

The selection of the study area is critical to ensure accurate data fitting and measurement; thus, it has to meet the following standards:

1. To ensure that the logic of urban development is as similar as possible, the cities in the study area should have similar contexts and development policies
2. To ensure the continuity of population data, the study area should have a homogeneous distribution of population in the region and a strong hierarchical structure of cities
3. To avoid statistical errors, there should be no overpopulated and undersized cities in the study area

Given these prerequisites, Shandong, China was chosen to be the target region in this study, which is located along the east coastline in China. Compared to other provinces, the cities of Shandong province have smaller gap in development but stronger hierarchical structure in size, which guarantees a more consistent data sample.

**Fig 1:**
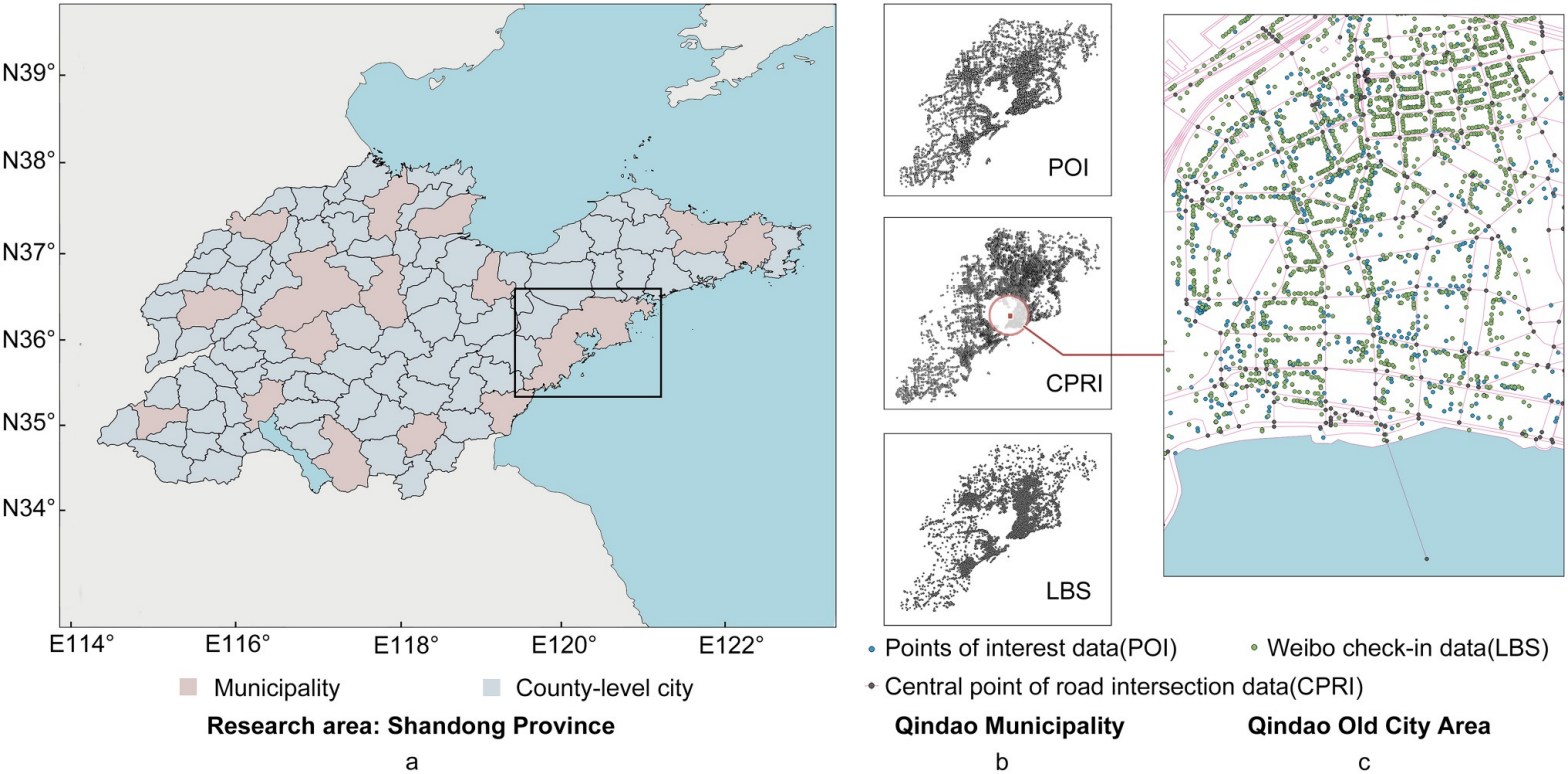
Visualization of the datasets used in this study. a) The division of Shandong province into 95 cities; b) Spatial pattern of three types of datasets of Qingdao; c) Spatial pattern of all the datasets of Qingdao. © OpenStreetMap [18] contributors.

According to the urban administrative and geographic city system of China, we identified 95 cities in this study, which include a total of 16 prefecture-level and 79 county-level cities (see Fig 1a). A prefecture-level city, which is an important jurisdiction in city division, is composed of multiple county-level districts and cities. A municipal district, also called municipality, is the center and primary city in a prefecture-level city, and consists of the administration departments of the prefecture-level city. The study guarantees spatial consistency by using the terms municipality and prefecture-level city to define cities. In some special cases, we address them separately:

1. The jurisdiction of Qingdao includes Jiaozhou, which is considered as part of the downtown area in the Qingdao master plan regardless of its county-level city status, and is closely connected with Qingdao’s jurisdiction geographically. Therefore, in this study, it is included in the statistics of Qingdao’s jurisdiction.
2. Similarly, Hengtai is regarded as a part of Zibo’s downtown area.
3. The city of Laiwu was an independent prefecture-level city, but was brought under the jurisdiction of Jinan City in 2019. To maintain consistency with other datasets, these two districts were calculated separately.

### Data

#### Central Point of Road Intersection data(CPRI)

The OSM was used as our road dataset, which is open source and contains data up to the end of 2018. We extracted the central point of road intersections of this dataset using ArcMap, a geospatial processing program. Approximately 273,000 points of road intersections were generated, and divided by related jurisdiction. Subsequently, we separately extracted the road intersection dataset of each of the 95 cities.

#### Points Of Interest data(POI)

Gaode Maps is China’s leading location-based services (LBS) provider of digital map content and navigation. Its open-source platform provides convenient information collection tools for academic purposes. The data used in this study is point of interest (POI) from the dataset up to the end of 2018, which contains approximately 668,000 point-form records; these are not only a direct representation of the underlying social network, but also serve as nodes of multiple productive activities.

#### Weibo check-in data(LBS)

With the prevalence of internet and domestic communication technology development, as well as the widespread usage of smartphones, many citizens in China are sharing their daily activities with a domestic microblogging service, named Weibo. Internationally, social media, such as Facebook or Twitter, have been widely used to reflect people’s routes in previous social science researches. Although Weibo provided an official API platform, it was closed in 2014. However, we can still access the historical data of 2014. The check-in data shows the places where users post their Weibo, which illustrates citizens’ activities and corresponding locations at certain time slots.

#### GDP data from statistical yearbook

Shandong Statistical Yearbook 2019 is an annual statistical publication that comprehensively reflects the economic and social development of Shandong, China. It covers data for 2018 together with key statistical data in recent years. The urban–-rural gap is the most important parameter to reflect China’s economic and social development. The urban–rural disparity cannot be necessarily narrowed with economic growth, but rather is an outcome of the interaction of resource allocation distortion, biased income distribution, and imbalance in inter-sector technological progress. From the economic chapter, we can collect urban and rural economic information separately, which can clearly describe the urban–rural gap quantitatively.

Fig 1b and 1c take Qingdao as an example to geographically display all these three point-form data. Furthermore, we built a statistical model to explore these data.

### Network distance statistical model

In this study, a complete weighted graph was used to represent an urban system, in which the edge weight is the Euclidean distance of the geographically projected nodes. We first obtained the projection coordinates of each point data, and then used the Pythagorean theorem to calculate the relative distance between all node data. This process generated a large amount of numerical data. Then, we drew a histogram of the frequency distribution based on the data. When the data size reaches a certain amount, the quantitative relationship of frequency can be seen evidently; this relationship demonstrates the different distribution laws of various node types. Python and Mathematica are used for calculation and visualization respectively. In this way, we compared their statistical characteristic values, such as mode and average, with the populations. Here, these statistics describe the degree of network connectivity. For cities, the relative distance represents the characteristics of the urban layout. Following logical steps, after data processing, we used existing statistical models to fit it. A log-normal distribution model was employed, which displays a normal distribution in the logarithmic scale.

In addition to comparing with population data, we further examined the impact of urban layout structure on urban size with economic data. Based on the observation of the data form, we investigated the correspondence between spatial inequality and urban structure, as well as the inherent logic. During processing, the population connects these two variables. All data were standardized using standard cities to observe the relationship between various parameters. We defined the coefficient *k* of the relationship between mode and population and observed its characteristics, as well as the impact of this coefficient on economic development. Through the definition of *k*, the structure of the city can be quantified to establish a relationship with the population.

## Results

### Spatial distribution of urban elements and causes

**Fig 2:**
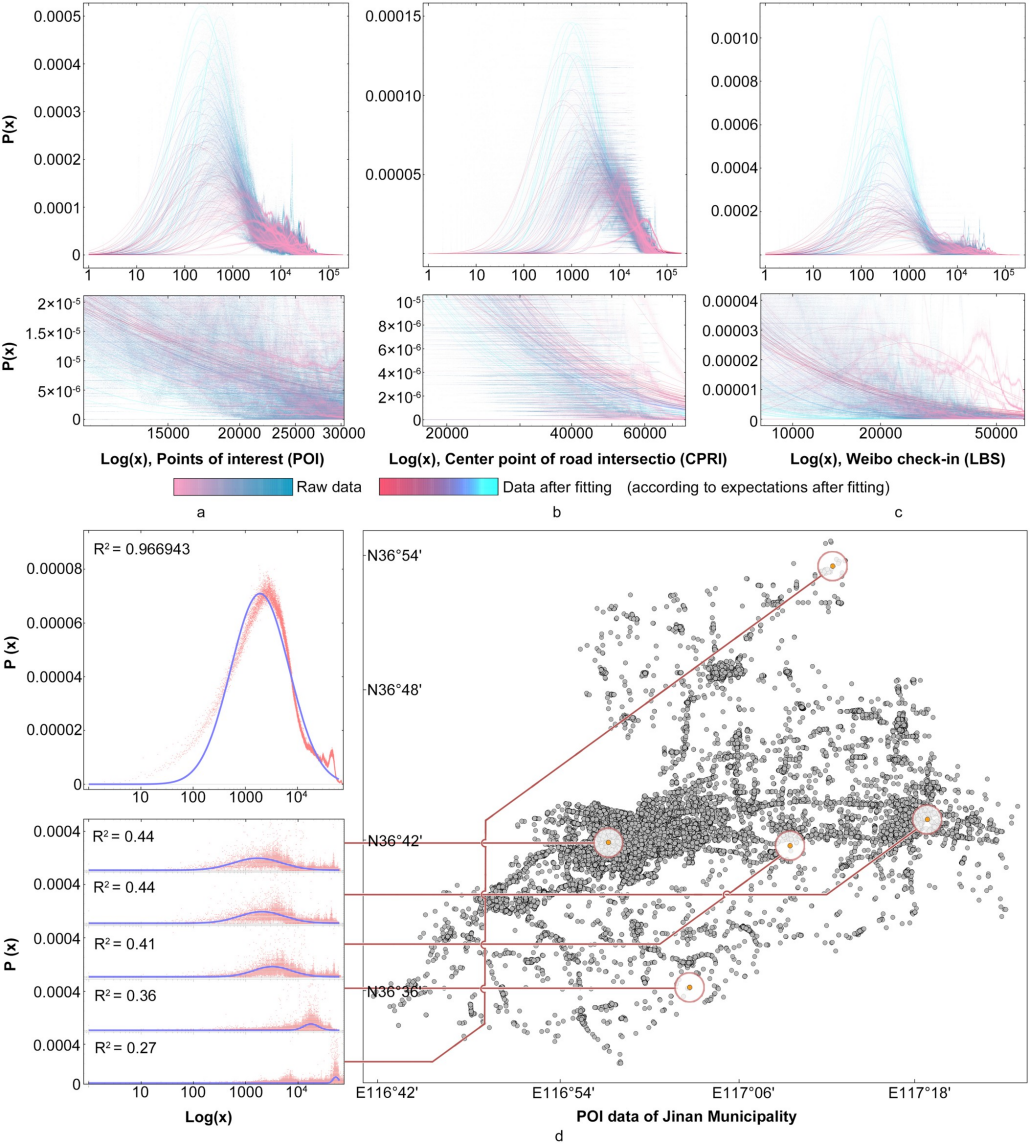
Visualization of data statistics. a) *Log*(*x*) of POI; b) *Log*(*x*) of the center points of road intersections; c) *Log*(*x*) of LBS; d) *Log*(*x*) of POI data and spatial pattern of Jinan Municipality.

Based on these statistics, we observed that the frequency distributions of different distance values share similarity in ascending order. In other words, they all demonstrated a type of skewed distribution, which fits well with the log-normal distribution model. The statistical result of 16 prefecture-level cities are illustrated Table 1, while the data of 95 cities are presented in S1 Table. According to these statistics, it can be stated that the fitted variance above 0.8 accounts for 92.63% of the overall results, while that above 0.9 accounts for 63.16%, which indicates a good fitting effect. The three different types of original data and corresponding fitted curve in the coordinate system are shown in Fig 2a–2c. To better illustrate the peak state distribution of the data, we assigned different colors according to expectations after fitting, and processed the data using a log-linear coordinate system to ensure the normal distribution of data. The detailed diagram at the bottom shows the tail state of the distribution.

**Table 1:** Statistics results of 16 prefecture-level cities.

City	Population (10, 000 people)	Area (m^2^)	Number			Before fitting (m)		After fitting (m)		*R*^2^
			POI	LBS	CPRI	Mode	Average	Mode	Average	
Bincheng	62.75	1040	5177	4901	2443	677.531	4101.07	731	5534.31	0.98
Dezhou	125.19	1751	14966	13112	3137	803.768	8700.25	1051	12381	0.97
Dongying	112.64	5776	10853	20429	3509	959.573	8700.45	581	22241	0.79
Heze	239.87	2261	11776	9211	3729	878.83	7984.67	801	13055	0.95
Jinan	540.58	6118.9	78826	62193	28568	1903.84	16990.64	1810	9156	0.97
Jining	189.05	1534	16785	18128	2765	1903.84	12178.75	1810	7981	0.94
Liaocheng	185.48	2446	20084	5998	6028	1097.92	27628.69	1131	42887	0.86
Linyi	281.18	2294	21596	18880	29112	1763.23	24953.33	971	12104	0.98
Qingdao	604.24	6549.6	128366	91933	40032	7748.78	23523.73	3691	41056	0.88
Rizhao	143.45	2046	15093	9494	4458	1287.54	8463.34	1701	9156	0.97
Taian	172.06	2087	11497	19083	5950	839.313	5980.39	1001	8382	0.99
Weihai	135.85	2607.7	21114	25463	7178	909.745	44201.82	731	33024	0.88
Weifang	186.39	2633.6	29625	22588	11141	1697.67	5949.98	1641	8410	0.96
Yantai	196.41	2864	35515	20406	5333	1103.26	7125.07	1859	13608	0.91
Zaozhuang	247.36	3068.8	13357	14002	9462	416.538	17655.56	831	98347	0.62
Zibo	339.39	3498.4	34637	33595	5737	1909.44	17189.58	1111	55133	0.77

POI: points of interest data, LBS: Weibo check-in data, CPRI: central point of road intersection data

In conclusion, the rule of internal node layout of a city is that the probability of the Euclidean distance between any two nodes exhibits log-normal distribution. Thus, we can employ the probability density function of the log-normal distribution to reveal the corresponding distribution law (Eq (2)):

$$\begin{array}{c} P(x)=\frac{e^{-\frac{{\left(\text{log}\left[x\right]-\mu \right)}^{2}}{2\sigma^{2}}}}{x\sigma \sqrt{2\pi }} \end{array}$$ (2)

where *μ* and *σ* represent two parameters, and x indicates the target object of analysis. In this study, x (unit: m) refers to the relative Euclidean distance of the three data types (points of interest, Weibo check-in data, and central point of road intersection). The distribution exhibits the following characteristics: in the interval where the relative distance is relatively small, the probability rapidly increases to the peak, and then declines gradually as the distance continues to increase, with the rate of decline exhibiting a reducing trend. From an empirical perspective, this feature is in line with the distances of human travel habits.

Mathematically, because the relative distance from one node to other nodes conforms to the skewed distribution of different characteristics, and coupled with a large number of nodes in the central area of the city (where the nodes are most densely clustered), the peak of these nodes will be close to the y-axis. The fact that the probability distribution of the distance from a certain node to other nodes is a subset of the overall distribution leads to the superimposition of a large amount of data in the central area, as well as makes the whole peak to be closer to the y-axis, thus demonstrating a logarithmic normal distribution pattern. It can be deduced that if a node is in the center of the city, and there exists no upper limit on the distribution density, then we can expect this probability to reach an exponential distribution, which is the limit of the spatial distribution of the nodes. Another limitation is that if a point is on the edge of the city, its related distances should exhibit normal distribution. As the distance between the nodes and city center increases, the corresponding peak transforms from logarithmic into normal distribution. Finally, the results of all nodes accumulate to form a log-normal distribution. In Fig 2d, the statistics of Jinan are taken as an example to verify this speculation. A total of 5 nodes were selected in different locations of the city, and the probability distribution of the distance between each node and the other nodes is calculated using a log-linear coordinate system. It can be observed from the graph that the peak of the outermost node in the city is the farthest from the y-axis, which approaches the city center as the node becomes closer to it, thus improving the fitting effect. As a result, all data distributions demonstrate a normal distribution in log-linear coordinates, allowing the corresponding log-normal distribution model to achieve the best fitting effect.

### Relationship between the urban internal node layout and the city size

**Fig 3:**
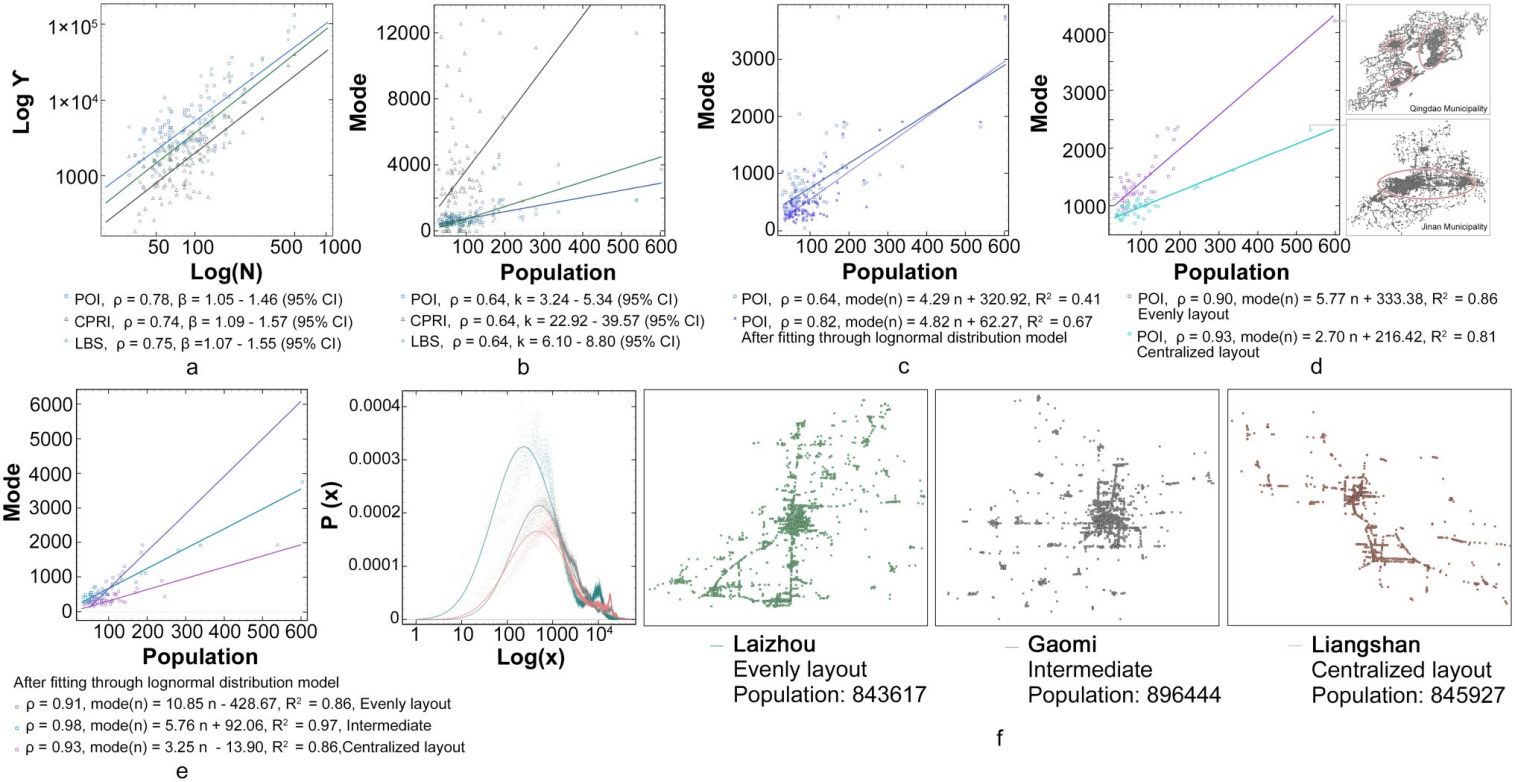
Relationship between internal node layout and the city size. a) Shape of three data types after model fitting; b) Plot of relation between datasets and population under 95% confidence interval; c) Correlation between the mode of POI data and population before and after the log-normal distribution fitting; d) Two classification results of the POI data; e) Three classification results of the log-fitted data; f) Curve of data in log-linear coordinates and spatial patterns of the three cities.

In addition to summarizing the statistical characteristics of the internal node layout of the city, we further investigate its correlation with the city size, which proves to be a power law between the total number of data Υ and the city size. The correlations of three different data types become significant at the level of 0.01, with all the Pearson coefficients *ρ* being above 0.7, leading to a high correlation. The three datasets and their fitted shapes are illustrated in Fig 3a. Under the 95% confidence interval, the *β* of the POI data is 1.05–1.46, road intersection data (CPRI) is 1.09-1.57, and the LBS data is 1.07-1.55, which is consistent with the law of city size.

From Table 1 and S1 Table, we can observe that the statistical characteristic values of the log-normal distribution exist in the internal node layout, aiding to observe the relationship with the city size. By statistical analysis and information comparison, we find that the modes of the three data types exhibit a significant relationship with the city size at the level of 0.01, with all the correlation coefficients *β* being 0.64, which is a medium correlation. This indicates that there exists a positive correlation between the data distribution of these three types and the city size. In terms of the human behavior pattern, this mode could represent the distance in the network. In behavior analysis, the highest frequency behavior is always more representative than the average or other characteristic values. Therefore, the slope between the data and city size is defined as *k*. According to Fig 3b, under the 95% confidence interval, *k* of the POI data is 3.24–5.34, road intersection data (CPRI) is 33.92–39.57, and the LBS data is 6.10–8.8. The comparison of the correlation between the mode of POI data and the population before and after the log-normal distribution fitting is illustrated in Fig 3c. To better reflect the possible characteristics of the overall urban system, the impact of outliers on the overall data can be decreased by fitting. Therefore, the correlation with the population size is stronger. However, the wider value range of *k* indicates the instability of the data.

To reduce such data instability and improve the effectiveness of fitting, the degree of data dispersion is related to the spatial structure of the city. For example, areas where large numbers of nodes gather result in data dispersion of different degrees. Therefore, cities can be classified based on their spatial structure, which can greatly improve the fitting effect. The classification results of the original POI data are presented in Fig 3d. In this study, the data are categorized into two layouts, namely, even and centralized, with individual slopes *k*. The correlations of both are above 0.9, and the best fitting effect is achieved once the variances reach above 0.8 after fitting. The comparison of Jinan and Qingdao shows this great difference, wherein Jinan demonstrates a distinctive single-center structure, while Qingdao apparently exhibits multiple centers.

The classification results of the log-fitted data are plotted in Fig 3e. Here, to achieve better fitting effect, the data are categorized into three layouts, namely, even, intermediate, and centralized. According to these three categories, we take three cities with similar population sizes (Laizhou, Gaomi, and Liangshan) as examples. The performances of these three cities in the log-linear coordinate system and the corresponding geographic distribution of the urban space are demonstrated in Fig 3f. Among them, Laizhou has many urban centers, and thus, presents more small concentrated areas, resulting in a more uniform layout picture as a whole. Although Gaomi has a single urban center, multiple small-scale concentrated areas exist in its countryside. Compared to the Gaomi urban structure, the rural areas in Liangshan are highly underdeveloped; consequently, the gap of urban–rural dual structure is large, resulting in a small *k* value.

In summary, the internal layout of cities with the same *k* value has similar logic, which enables the feature values (modes) of these urban data to be scaled according to population. Moreover, *k* can measure the logic of the internal node layout of the city feature. The relationships between the urban structure and *k* can be described as follows:

1. More concentrated urban spatial layout leads to a smaller *k* value;
2. More uniform urban spatial layout leads to a greater *k* value;
3. For cities with multiple centers and similar scales of size, the geographical scale of their elements is larger, with better *k* value performance than those of cities with a single center.

### Performance of internal node layout rules in urban power law

**Fig 4:**
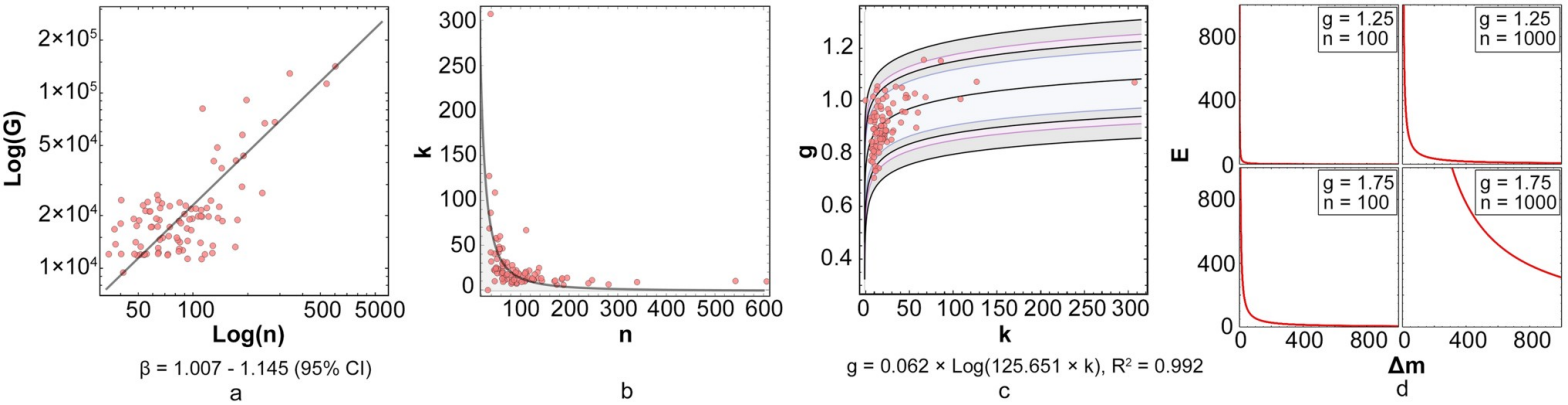
Relation of internal node layout rules by urban power law. Plot of a) urban–rural income gap and population, b) *k* value and population, c) conversion rate *k* of the mode and power coefficient *g*, and d) function under two different Δ*n* and *g*.

An analysis of the results indicate that a positive correlation exists between the mode of the relative distances and the population. However, this correlation coefficient is not fixed, and the mode of a city with larger population is not always bigger than that of a less populated one. This can be explained by the fact that different cities have unique internal network structures, resulting in various correlation coefficients. Accordingly, cities with similar structures have similar correlation coefficients. For a single city, the coefficient can be regarded as the conversion rate of the mode with the population. As in the above-mentioned law of city scale, many phenomena in cities are related to the city scale in a linear or hyper-linear manner. Because the law of urban scale can be related to the distribution law of urban spatial elements, the performance of urban spatial elements in the law of city scale is further investigated. In the previous comparison of the three cities (Laizhou, Gaomi, and Liangshan), we found that the small urban coefficient value of Liangshan was due to the insufficient development of its rural areas. Therefore, in this section, the focus is on urban inequality. Specifically, we explore the relationship between the urban–rural income gap and the internal network structure of the city. The urban–rural gap is an important issue during urbanization in China. We take the difference of the disposable income of urban and rural areas from the statistical yearbook to quantify this gap. The relationship between the urban–rural income gap and population is illustrated in Fig 4a, which basically conforms to the urban power law.

Because each city has a different spatial form, they have their own mode-to-population conversion rate (denoted as *k*), *k* = Δ*m*/Δ*n*, which can be used to measure the degree of dispersion and concentration of nodes within the city. To compare all cities, we selected a certain city as our standard, and standardized the data of all cities based on it. A city can be compared with the standard city using Eq (3):

$$\begin{array}{c} k=\frac{m-m_{s}}{n-n_{s}} \end{array}$$ (3)

Therefore, the relative conversion rate of the city can be obtained. In the above equation, *m*_*s*_ and *n*_*s*_ represent the mode and population of the standard city, respectively. In the subsequent research stages, to avoid negative value generation, we chose Qingyun, the smallest city, as the standard city, i.e., *k* = 1 is assumed for this case. Through statistics, we also observed that an exponential relationship exists between the *k* value and population of a city (as shown in Fig 4b, the index is -0.74), while *k* was concentrated in the middle with a small number of tails.

Similarly, each city has its own transformation rate of the urban–rural income gap with population. In this study, we use the urban–rural income gap and the power coefficient of population (denoted as *g*) to measure the growth rate of urban–rural gap with population. The relationship between population and urban–rural income gap can be expressed by *αn*^*g*^ = *G*, where *G* represents the urban–rural income gap, while a is the standard coefficient that can be obtained by setting a standard city. Here, we assume that Qingyun’s urban–rural income gap is proportional to population, i.e., *g* = 1 for Qingyun. Using Qingyun’s data of income gap *G*_*s*_ and population *n*_*s*_, we can calculate the parameter *α* = *G*_*s*_/*n*_*s*_, and obtain the power coefficient for each city’s urban–rural income gap and population using Eq (4).

$$\begin{array}{c} g={\text{log}}_{n}\frac{n_{s}G}{G_{s}} \end{array}$$ (4)

In this way, we can associate the urban–rural gap with the characteristic value of the urban network through population. As shown in Fig 4c, a logarithmic distribution exists between the conversion rate *k* of the mode and the power coefficient *g* of the urban–rural income gap. It can also be observed from the same figure that the performances of the fitting logarithmic function under the confidence intervals are 80%, 90%, 95%, and 99%, respectively. Therefore, there exists a logarithmic relationship between *k* and *g*; in other words, if the length mode of the internal network connection of a city with a certain population decreases and causes *k* to decrease, it will further reduce its g, which in turn can narrow down the urban–rural income gap in the city. In addition, according to the nature of the logarithmic distribution, when *k* is large, i.e., when the internal node layout of the city is more uniform, the effect of reducing the mode and improving the urban–rural gap through adjustment of the internal node layout of the city is small. Contrarily, if *k* is small, i.e., the layout of nodes in the city is more concentrated, and reducing the mode can have a better improvement effect. A larger *k* value corresponds to more uniform distribution of internal nodes, and thus, narrowing down the gap between urban and rural areas becomes more challenging, while facilitating the effect of length mode reduction of the internal network connection in a concentrated city.

Moreover, we can summarize the control efficiency of the urban–rural gap. We use the conversion amount ratio of the urban–rural income gap to the mode to define the utility (see Eq (5)):

$$\begin{array}{c} E=\frac{\Delta G}{\Delta m}=\frac{g\Delta n^{g}}{k\Delta n} \end{array}$$ (5)

That is, for every 1 m increase in the mode, the income gap increases by E million Chinese Yuan (CNY). In the formula, *k* can be replaced by Δ*m*/Δ*n* to establish a relationship between the mode and utility. Then, we can further obtain Eq (6):

$$\begin{array}{c} E=\frac{g\Delta n^{g}}{\Delta m} \end{array}$$ (6)

In this formula, Δ*n* represents the possible population change; Δ*n* and *g* are two parameters, while Δ*m* represents the possible change in the network structure. Using this efficiency formula, we find that no relationship exists between the trend of utility change with Δ*n* and *g*, while Δ*m* and *E* are related to the power of -1. Larger mode change corresponds to lower utility, making it more challenging to improve the urban–rural gap, and allowing the utility to decrease faster in the closer interval. Here, *g*Δ*n*^*g*^ can be considered as a utility coefficient, whose specific role varies depending on the conditions of Δ*n* and *g*. From the perspective of urban structure, this suggests that controlling the differentiation of urban–rural gaps becomes increasingly difficult during urban development. The shapes of the function under two different Δ*n* and *g* are illustrated in Fig 4d.

The above series of statistical research and quantitative concept definitions can be extended to the classical city power law, i.e., the power relationship between GDP and the city size. We use GDP data to quantify the development gap between the three cities. First, we explain the relationship between population growth and GDP increase from the perspective of the utility formula. For a certain city, its g and k parameters are also determined, with *g* being approximately 1.2 in general. Thus, based on the utility formula Eq (6), we can further obtain Eq (7):

$$\begin{array}{c} E=\frac{g\Delta n^{g-1}}{k} \end{array}$$ (7)

According to this, larger population increment results in greater utility, where a power function relationship exists between these two parameters, with the power value being less than 1. This reflects the stimulating effect of population on the urban–rural gap. If cities in the same area are assumed to have the same power coefficient g, then the utility *E* and the value of *k* are apparently negatively correlated during a certain population increase. This demonstrates that more concentrated cities may have more potential room for improvement, and a more uniform urban structure corresponds to lower utility.

**Fig 5:**
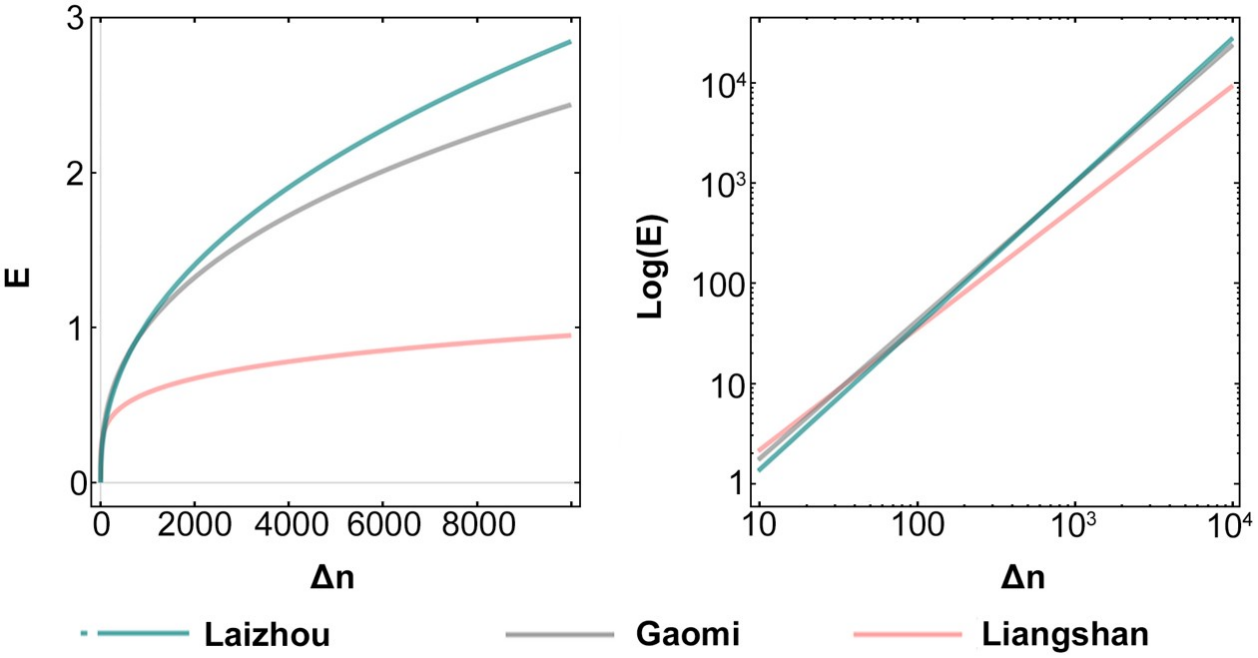
Relationship between the increase in population and GDP of Laizhou, Gaomi, and Liangshan.

The relationship between the increase in population and GDP of the three cities with similar above-mentioned population is presented in Fig 5. We can easily observe that the GDP increase is positively correlated with the population growth, while its increasing rate decreases as the population continues to increase. The curves corresponding to the three cities also show their different utilities. This utility echoes the value of k, which proves the influence of *k* on urban development. Further, from a certain perspective, this explains the fact that cities within the same region, even with similar development conditions and higher-level planning, may still have differences in GDP under the same population size.

## Discussion

In this study, to obtain the statistical law of internal node layout, the urban system was regarded as a complete graph weighted by Euclidean distance. Through the statistics and analysis of multiple datasets, the relative Euclidean distance between certain point data (points of interest, Weibo check-in data, and central point of road intersection data) presented a log-normal distribution, and its mode was positively correlated with the population size. The mode can be scaled according to the population size in different cities. In this scaling, the clustering of correlation coefficients caused by similar urban structures was significant, which indicated the possible existence of logic between the urban structure and the slope *k* of the mode and population, and thus, verified the importance of *k* in urban structure identification. From this point of view, it can be stated that each city has its own network form and scaling factor accordingly. Therefore, by selecting a standard city to compare with all other cities, the study explored the influence of the network structure of city nodes on the power law in the city. This study specifically confirmed the relationship between the changes in urban node network structure and urban inequality. Therefore, there exists a logarithmic relationship between the conversion rate *k* of the weight of the network edge with the population scaling and the urban–rural income gap with the power coefficient *g* of the population. On this basis, this study further investigated ways to narrow down the gap between urban and rural areas through urban structure adjustment. In addition, the laws obtained in this study can be expanded to explain other phenomena that conform to the law of city size; for instance, why GDP may vary among cities even with the same population size.

Log-normal and similar distributions have been studied previously, with various studies focusing on the urban order distribution [19–21]. Further, other variables have also been introduced, such as distance and landscape form, to more comprehensively describe the distribution law of a city [22, 23]. In many modeling and prediction of natural and man-made phenomena, the log-normal distribution model has been widely used as a classic probability distribution model [24–26]. In other words [27–29], it is continuously gaining popularity in studying human activities. A commonly accepted view is that a power law relation (1), whose distribution is derived from complex multi-objective optimization [30], exists between multiple variables and city size. Therefore, the corresponding log-normal distribution of distances is caused by such complex multi-objective optimization.

Currently, multi-centralization is a trend of urban development, which also makes cities increasingly more complex [31, 32]. By exploring the advantages and disadvantages of this trend, cities can effectively cope with changes during development. This study proved that although the urban–rural inequality gap could scale up as the population increases, there still exists a large difference even under the same population size. Even having the same population size, cities with a more uniform urban structure tend to have a smaller urban–rural income gap. Although the urban–rural gap of cities with concentrated structures was large, the potential for improvement is correspondingly large. Statistical conclusions and previous research results [33] are consistent with the history of urban development [34, 35]. Although the urban structure has been used to explain these statistical phenomena, other influencing factors remain to be investigated [36]. The logic of these statistical phenomena, the inter-relationship of parameters, and the dynamic mechanism of change still require further research.

It should be also noted that the obtained conclusion is a macro law based on a large amount of city data; in fact, specific cities need separate analyses. Moreover, the entire research was based on the current state, i.e., *k* was only the conversion factor of the current state, which may change with the development of the city. For a developing city, urban population growth results in potential changes in its associated parameters, leading to a strong uncertainty. For example, once the urban population increases, the number of network nodes increases accordingly, which may affect the structure of the entire network and its *k* value. A recent research confirmed the relationship between urban development and the law of urban size using long-term datasets [37]. Therefore, in future research, the time dimension can also be introduced to describe the relationship between the urban structure and law of urban size. The aim is to more comprehensively describe the changes in urban structure during urban development to simulate the dynamic process of urban space development. Based on this, our research results can serve as the basis of dynamics.

## Conclusion

Based on three different spatial data, this study verified the relationship of city size and structure. Through statistical analysis and parameter definition, the impact of urban structure on regional inequality is quantified. The following conclusions are drawn:

1. The probability that the distance between all nodes in the city conforms to the log-normal distribution in ascending order, and the distance of the highest probability value (i.e., the mode of the relative distance) is positively related to the size of the city.
2. Taking a certain node in the city as the starting point, as the distance increases, the probability of other nodes appearing geographically conforms to a kind of off-peak distribution, which is in between the log-normal and the normal distributions. The location of the peak is related to its geographic position.
3. Due to the linear correlation between the mode of relative distance and the population, we defined its conversion rate *k* as an indicator to quantitatively describe the urban structure; it can better explain the correspondence between the statistical data and the urban network layout.
4. By comparing the relative distance mode, population conversion rate *k*, urban–rural gap, and population power parameter *g*, we investigated the relationship between the urban–rural gap and the mode, and defined the utility formula using the mode to narrow down the urban–rural gap, which can explain the difficulty in narrowing the urban–rural gap in cities, and the relationship between the urban structure and size.
5. We deduced the utility formula between urban development and urban structure. The utility formula can be applied to address regional inequality, and explained the differences in the urban development of cities with similar sizes from the perspective of urban structure. The derivation results prove that, in the case of the same population size, the urban structure that is scattered at the macro level and concentrated at the micro level has greater development potential.

To summarize, this study achieved statistical laws existing in the layout of urban spatial elements and developed an approach to quantify the features of urban structures. It provided a new perspective of urban structure to understand the complexity of a city.

## Supporting information

S1 Table Statistical results of 95 cities. Detailed statistical values of the 95 cities in Shandong (CSV).
(XLSX)

S1 File (RAR)
